# Seroprevalence of bovine brucellosis and associated risk factors in and around Alage district, Ethiopia

**DOI:** 10.1186/s40064-016-2547-0

**Published:** 2016-06-23

**Authors:** Hagos Asgedom, Delesa Damena, Reta Duguma

**Affiliations:** National Animal Health Diagnostic and Investigation Center, P. O. Box 04, Sebeta, Oromia Ethiopia; Department of Clinical Studies, College of Veterinary Medicine and Agriculture, Addis Ababa University, P. O. Box 34, Bishoftu, Oromia Ethiopia

**Keywords:** Alage, *Bovine brucellosis*, Seroprevalence, Risk factors, Intensive and extensive farms

## Abstract

Brucellosis is a zoonotic disease with economic and public health impact, particularly for human and animal populations within developing countries that relay on livestock production. A cross sectional study was conducted between October 2013 and March 2014 in and around Alage district to determine the seroprevalence of bovine brucellosis and associated risk factors. A total of 804 sera samples; 421 from cattle managed under extensive production system and 383 from cattle managed under intensive production system were collected. Multistage cluster sampling method was employed to sample unvaccinated cattle above 6 months of age. Rose Bengal Plate Test and c-ELISA were used in serial for detection of antibodies against *Brucella* species. The overall seroprevalence was 2.4 %, and herd level seroprevalence was 45.9 %. A prevalence of 3.3 and 1.3 % was recorded in the extensive and intensive farms respectively. Among the three sites, seropositivity of 3.4 % in Naka, 3.3 % in Negelewudisha and 1.3 % in Alage were recorded. Risk factors such as age, sex, number of service per conception, calving interval and reproductive status were associated with serostatus of brucellosis. Taken as a whole, cattle in both intensive and extensive production systems are endemically infected by brucellosis at low level in the study areas. This warrants the need of integrated intervention strategies to minimize the spread of the disease in animals and reduce the risk of transmission to humans.

## Background

Brucellosis is a highly contagious, zoonotic and economically important bacterial disease of animals worldwide and it is considered as one of the most widespread zoonoses in the world (Schelling et al. 2003). The disease in cattle, usually caused by *Brucella abortus* and occasionally by *Brucella melitensis* and *Brucella suis*, is characterised by late term abortion, infertility and reduced milk production (OIE 2008). Aborted foetuses and discharges contain large number of infectious organisms and transmit the disease within and in between herds. In addition, chronically infected cattle can shed lower numbers of organisms via milk and reproductive tract discharges, and can also vertically transmit infection to subsequently born calves, and maintain disease transmission (McDermott and Arimi 2002). In dairy production, the disease is a major obstacle to the importation of high yielding breeds and represents a significant constraint to the improvement of milk production through cross breeding (Mustefa and Nicoletti 1993).

The epidemiology of cattle brucellosis is influenced by several factors including factors associated with disease transmission between herds, factors influencing the maintenance and spread of infection within herds (Crawford et al. 1990). Understanding the epidemiology of brucellosis is therefore, vital for strategizing evidence based disease control measures. However, such information is inadequate in sub-Saharan Africa. Consequently, appropriate preventive measures have not been undertaken in this part of the world (McDermott and Arimi 2002).

In Ethiopia, there is no documented information on how and when brucellosis was introduced and established. However, several serological surveys have showed widespread distribution of brucellosis in different livestock production systems (Dinka and Chala 2009). For instance, a seroprevalence of 2.4 % in Jimma zone (Tolosa et al. 2008), 4.2 % in south east Ethiopia (Bekele et al. 1999), 2.9 % in central Oromia (Jergefa et al. 2009), 11.2 % in east Showa zone (Hunduma and Ragasa 2009) 4.9 % in Tigray region (Hileselassie et al. 2008) were documented.

However, these studies were geographically limited and didn’t provide adequate information on risk factors that play role in occurrence and transmission of the disease. Particularly information is lacking in intensive farms although few scattered data are available. Therefore, the present study attempts to elucidate the sero-prevalence of brucellosis in intensive and extensive farms and assess associated risk factors in cattle in and around Alage district aiming at providing inputs for evidence-based disease control in Ethiopia.

## Methods

### Description of the study area

The present study was conducted in Alage Agricultural Technical and Vocational Education Training (ATVET) College and two surrounding Peasant associations (PAs). Negele Wudisha PA is located south west of Alage in Alaba district, Southern Nations and Nationalities regional state. Naka PA is located at North east of Alage in kombolcha district, Oromia regional state. The districts are situated at 217 kms South West of Addis Ababa at longitude of 38°30′East and altitude of 7°30′North with an altitude of 1600 above sea level. The area is characterized by mild sub tropical climate with average minimum and maximum temperature of 11 and 29 °C respectively. There are two defined rainy seasons: short rainy season (March–April), long rainy season (June–September). These two PAs have high cattle population compared to other PAs in the area. The total cattle population in both PAs is estimated to be 7, 550 (ARDOAD 2012).

### Study animals

The ATVET Collage intensive production system consisted of four separate herds each containing more than 200 heads of cattle. Two of the farms contained Borana breeds and the remaining two herds contained Holstein cross breeds. The Holstein cross breeds are kept for milk production whereas the Boran breads are kept for fattening.

The extensive system consisted of Arsi cattle breeds reared under traditional small holder production system. In small holder farming, cattle form an integral part of the people’s livelihood, provide a vital source of milk, meat, income, draught power for land tillage, transport and manure in addition to their use in numerous social and cultural roles. Cattle above 6 months of age including milking, none milking, replacement heifers and bulls were included in the study.

### Sample size and sampling strategy

Herds were regarded as the primary sampling units and stratified according to the study area. In each study area, the approximate numbers of herds were listed with the assistance of local animal health office and community leaders. The herd was categorized into two groups based on the prevailing herd size in the study area as small (<20) and large (>20). Those herds that were reared in close proximity of each other (housed in consecutive barns and graze on the same field) were grouped together and considered as one although owned by different farmers. Only herds with a minimum of 10 cattle that were older than 6 months at the time of sampling were included in the study. The list of herds was obtained from animal health office and community leaders. Out of a total of 78 herds, 16 small herds and 17 large herds were randomly selected in both study districts; all the four intensive farms were sampled.

Based on previous validation studies, the diagnostic sensitivity and specificity of Rose Bengal Test (RBT) were assumed to be 90 and 75 %, respectively and for the competitive enzyme-linked immunosorbent assay (c-ELISA) 98 and 99 %, respectively (McGiven et al. 2003; Nielsen et al. 1995). Therefore, at individual animal-level, the combined sensitivity and specificity for RBT and the c-ELISA using a serial interpretation were calculated to be 88.2 and 99.8 %, respectively, as suggested by Noordhuizen et al. (1997). Based on these parameters, the sample size of individual cattle was estimated by using FreeCalc version 2 (Cameron 1999). Selection of cattle to be sampled from each herd was based on a systematic random sampling technique, where cattle were put in a crush pen and one animal was selected from five consecutive animals coming through the crush pen. A total of 804 cattle: 384 from the intensive farms and 420 from extensive farms were sampled.

### Sample and data collection

Approximately 10 ml amount of blood was taken aseptically from the jugular vein of each animal using plain vacutainer tubes and needles. Sera were kept at −20 °C in Alage ATVET animal health laboratory until serological tests were conducted. A questionnaire survey with open and closed questions was used amongst the owners whose animals were tested. The questions were specifically designed to get information from the animal owners and farm owners. The following data was collected on animal attributes: breed, sex, age, and reproductive status, stage of lactation, calving intervals and number of service per conception. Based on its biological relevance, age was stratified into three categories such as: 0.5–3, 3–6, and >6 years. The reproductive status was also categorized as heifer, pregnant, lactating and dry. Besides, information on farms such as: hygienic practices, herd size, and other managemental factors were collected. The presence of calving pens, waste (placenta, aborted material and dead animal) disposal methods was categorized into burying, open dump or feeding to dogs. Hygienic status of the farms was categorized as clean and not clean based on manure disposal, drainage and barn ventilation. Information about cause, symptoms, mode of transmission, prevention, and treatment was also collected to assess the awareness level of the farmers. Farmer’s knowledge on brucellosis was assessed using twelve closed ended questions which were then reduced to five items using principal component analysis. The internal consistency of the variables were also checked (cronbach’s α = 0.837). The respondents who scored above the mean rank were considered as having good knowledge about brucellosis and below mean rank were regarded as having poor knowledge.

### Rose Bengal Plate Test (RBPT)

The Rose Bengal Plate Test was conducted at the National Animal Health Diagnostic and Investigation Center, Sebeta (NAHDIC) for screening positive samples. Positive sera were then retested using enzyme linked immuno sorbent assay (ELISA). Samples were considered as positive for brucellosis, if they were positive for both RBPT and ELISA. The RBPT procedure was followed based on the description by Nielsen et al. (1995).

### Competitive ELISA

All RBPT positive sera were further tested using the SvanovirTM Brucella-Ab c-ELISA test kits (Svanova Biotech, Uppsala, Sweden) at NAHDIC. The test was performed according to the manufacturer’s instructions. The test was conducted in 96-well polystyrene plate (Nalge Nunc, Denmark) that were pre-coated with *Brucella* species lipopolysaccharide (LPS) antigen. Serum diluted 1:10 was added to each well followed by equal volume of pre-diluted mouse monoclonal antibodies specific for a common epitope of the *O*-polysaccharide of the smooth LPS molecule. The reactivity of the mouse monoclonal antibody was detected using goat antibody to mouse IgG that was conjugated to horseradish peroxidase. Hydrogen peroxidase substrate and ABTS chromagen were developed for 10 min. The reaction was then stopped using 1 M H_2_SO_4_. Optical densities were read at 450 nm using Titertek Multiscan^®^ PLUS reader (Flow Laboratories, UK). The threshold for determining seropositivity was based upon the manufacturer’s recommendations (>30 %), with antibody titers recorded as percentage inhibition as defined by the ELISA kit supplier.

### Data analysis

Data was entered into Microsoft (MS) Excel Spread Sheet program and statistical analysis was computed using Chi-square test and logistic regression using SPSS 19 window evaluation version. The total prevalence was calculated by dividing the number of RBPT and c-ELISA positive animals by the total number of animals that were tested. Herd level prevalence was calculated by dividing the number of herds with at least one reactor in RBPT and c-ELISA by the number of all herds tested. The association between risk factors and seropositivity to *Brucella* species was considered as significant at P < 0.05. Odds ratio (OR) was used to measure the degree of association between risk factors and seroprevalence of bovine brucellosis.

### Ethics approval

All procedures were carried out according to the experimental practice and standards approved by the Animal Welfare and Research Ethics Committee at National Animal Health Diagnostic and Investigation Center (NAHDIC) that is in accordance with the international guidelines for animal welfare.

## Results

### Occupational risk and awareness among farm works and farmers about brucellosis

A total of 100 volunteer farm workers and herd owners were interviewed to assess the occupational risks and awareness levels. The majority of the participants in intensive farms were well aware of brucellosis. Up to 70 % of the respondents were regarded as having good knowledge about brucellosis. The level of awareness was significantly lower (P = 0.001) in extensive farms in which 78.3 % of the participants were regarded as having poor knowledge of the disease (Table 1). The greater parts (82.5) of the respondents in intensive farms utilize basic PPE on farm activities. However, only one-fifth of the farmers in extensive farms use basic PPE although the majority (78.3 %) of them had the experience of assisting animals giving birth. Besides, 70 % of the participants in intensive farms and up to 80 % those in extensive farms responded that they have habit of drinking raw milk or eating raw meat as shown in Table 1.

**Table 1:** Occupational risks and awareness among farm works and farmers about brucellosis in intensive and extensive farms in the study areas

Variable	Intensive farms (n)	Extensive farms (n)	*X* ^2^ value	*P* value
Knowledge about brucellosis				
Yes	28	13		
No	12	47	23.18	0.00
Use of PPE				
Yes	33	12		
No	7	48	37.88	0.00
Assisted animals giving birth				
Yes	21	47		
No				
Habit of drinking raw milk or eating raw meat	19	13	7.36	0.01
Yes	28	48		
No	12	12	1.32	0.34

In intensive farms, artificial insemination (AI) is used for breeding in herds with Holstein cross breeds and bull service is used in herds with Borana breeds. On the other hand, all herds in extensive farm use bull service for breeding. The farms in intensive farm were regarded as clean with good farm management practices. However, we observed poor hygienic practices including poor waste disposal, drainage and poor barn ventilation in the majority of the extensive farms.

### Seroprevalence of bovine brucellosis

In the present study, a total of 804 cattle sera were collected from 33 herds and four intensive farms which were not vaccinated against bovine brucellosis. Of them, nineteen (19) sera were positive with both RBPT and ELISA tests. An overall animal level seroprevalence of 2.4 % and herd level seroprevalence of 37.84 % were recorded. There were no significant variations of the seroprevalence among different farms, PAs and production system as shown in Tables 2, 3, and 4.

**Table 2:** Over all seroprevalence of bovine brucellosis in the intensive and extensive farms of the study area assessed by Chi-square

Factor	Level	n	Test +ve	%	OR	95 % CI	χ^2^	P value
Farm type	Intensive	383	5	1.3				
	Extensive	421	14	3.3	2.60	0.93–7.29	3.54	0.06

**Table 3:** Herd level seroprevalence of bovine brucellosis in intensive and extensive farms in the study area assessed by Chi square

Factor	Level	n	Test +ve	%	OR	95 % CI	χ^2^	P value
Farm type	Extensive	33	14	42.4				
	Intensive	4	3	75	4.07	0.38–43.38	1.52	0.22

**Table 4:** Seroprevalence of bovine brucellosis in three different sites in the study area assessed by logistic regression

Factor	Level	n	Test +ve	%	OR	95 % CI	P value
Sites	Alage^a^	383	5	1.3	–	–	–
	Negele Wudisha	246	8	3.3	2.54	0.82–7.86	0.105
	Naka	175	6	3.4	2.68	0.81–8.92	0.107

^a^In reference to

### Animal characteristics

There was a significantly low seroprevalence (0.4 %) of bovine brucellosis in animals with less than or equal to 3 years old compared to animals with greater/equal to 7 years old (5.3 %, P = 0.019). The logistic regression result also showed that older animals were 11.4 times at higher risk than younger animals. Seropositivity in females (3.4 %) was significantly higher (P = 0.007) than that of males (0.4). The females were 1.15 times at higher risk than males (Table 5). Besides, association between brucellosis and reproductive status of the cows was statistically significant (P = 0.001) by taking heifers as a reference (Table 5). There were no significant differences in seropositivity between breeds or between farm-sizes (Table 5).

**Table 5:** Association between animal characteristics and seropositivity of *Brucella* assessed by logistic regression and Chi-square

Factors	Level	n	Test +ve	%	OR	95 % CI	Z-test	P value
Age	0.5 ≤ 3^a^	233	1	0.4				
	3 ≤ 6	308	4	1.3	3.2262	28.32–30.53	1.0386	0.299
	≥7	263	14	5.3	11.376	90.33–85.47	2.3308	0.0198
Sex	F^a^	529	18	3.4				
	M	275	1	0.4	0.104	0.014–0.78	−2.3742	0.007
Breed	Arsi^a^	494	16	3.2				
	HF	149	1	0.7	0.1724	0.302–3.75	−1.3915	0.1641
	Boran	161	2	1.2	0.4001	2.02–4.15	−0.8571	0.3914
Herd size	≤20^a^	232	6	2.3				
	>20	572	13	2.6	1.6378	4.30–5.28	0.3586	0.7199

^a^Others were computed in reference to this category

### Association of *Brucella* seropositivity with reproductive performances of animals

Out of 29 animals that needed repeated services (more than or equal to three services), 10 cows (34.5 %) were seropositive for brucellosis. The logistic regression analysis indicated the strong association between brucella seropositivity and number of service per conception (NSPC) (P = 0.001).

Similarly, there was association between calving interval and seropositivity to brucellosis (P = 0.001). The seropositivity of cows that gave birth at an interval of 1 year, less/equal to 2 years and above 2 years were 2.1, 1.8 and 33.3 % respectively. The Odd ratio of *Brucella* seropositivity in cows that gave birth above 2 years interval was 27.62 times higher than those cows calving yearly (Table 6).

**Table 6:** Association between *Brucella* seropositivity and reproductive performance of cattle in the study area assessed by logistic regression

Factors	Level	n	Test +ve	%	OR	95 % CI	Z-test	P value
NSPC	1^a^	262	2	0.8				
	2	82	5	6	8.3974	41.96–46.23	2.5147	0.011
	≥3	29	10	34.5	62.053	303.4–311.55	5.0546	0.000
Calving interval	1 ≤ 2 years^a^	218	4	1.8				
	1 years	48	1	2.1	8.609	0.005–0.351	3.0178	0.003
	>2 years	33	11	33.3	27.622	0.011–0.127	84.683	0.000
Reproductive status	Heifer^a^	85	1	1.2				
	Lactating	140	2	1.4	0.821	0.073–9.20		0.87
	Dry	107	4	3.7	2.68	0.481–9.91	1.8413	0.26
	Pregnant	148	11	7.4	5.54	1.21–25.46	−1.996	0.028

^a^Others were computed in reference to this category

## Discussion

Knowledge and perception about brucellosis among farmers are crucial in controlling disease transmission. In this study, we interviewed farm workers and herd owners to assess their awareness levels about brucellosis and occupational risks using structured questionnaire. The participants in intensive farms were well aware of brucellosis. The majority (70 %) of the respondents were regarded as having good knowledge about brucellosis. We also demonstrated good hygienic practices; including separate calving rooms, use of clean water, regular cleaning of barns, proper waste disposal and use of PPE. The farm workers were regularly trained on biosafety practices by college instructors. Good knowledge about brucellosis has been shown to have a protective effect towards animal and human infections in Kyrgyzstan (Kozukeev 2003).

In contrast to this, the awareness level of the farmers was significantly lower in extensive farms. The majority (78.3 %) of the farmers were regarded as having poor knowledge. We also observed poor hygienic practices and uncontrolled animal movements. These could pose high risks of transmitting the disease within and in between the herds. This is in agreement with previous studies in extensive livestock production system in Ethiopia (Ragassa et al. 2009; Megersa et al. 2011). In addition to this, only one-fifth of the farmers in extensive farms use basic PPE although a big proportion (78.3 %) of them had the experience of assisting animals giving birth. The occurrence of brucellosis in humans is associated with contact with domestic animals (Alballa 1995), exposure to aborted animals and assisting animal parturition (Cooper 1992; Kozukeev et al. 2006). In this study, the majority of the participants in both types of farms have the habit of drinking raw milk or eating raw meat. Prevalence of brucellosis in humans is attributed to the culture and tradition of consuming raw milk and milk products (Omore et al. 1999).

The present study showed that the overall seroprevalence of bovine brucellosis in Alage and its surrounding was at low level (2.4 %). In extensive management system, a prevalence of 3.3 % was recorded. This is in agreement with the previous studies in different parts of Ethiopia in which a disease prevalence ranging from 1.5 to 3.5 % were reported (Megersa et al. 2011; Asmare et al. 2010; Berhe et al. 2007). However, the present result is lower than the reports of earlier studies in Abernosa cattle breeding ranch and East shoa where a seroprevalence of 19.5 and 11.5 % was reported by Yirgu (1991) and Dinka and Chala (2009) respectively. This study also showed a very low seroprevalence (1.3 %) of brucellosis in the intensive production system. This is in consistent with previous a study in and around Addis Ababa (Tefera 2006). On the other hand, higher seroprevalence ranging from 8.11 to 38.7 % were recorded in intensive production systems located in different parts of Ethiopia (Rashid 1993; Sintaro 1994; Gebremariam 1985). The observed disparity could be attributed to various factors including differences in testing protocols, cattle rearing systems, and herd size.

In the present study, a statistically significant association between sex and seroprevalence of brucellosi*s* was observed. Almost all (94.7 %) of the seropositive animals were females. This result was in agreement with earlier studies in Ethiopia where absence of male seroreactors was reported (Berhe et al. 2007; Tolosa 2004). The present study also showed the presence of statistically significant associations between age and seropositivity of brucellosis. This finding was supported by a previous report from Ethiopia (Asmare et al. 2010). Growth stimulating factors for *Brucella* organisms become abundant when the animal becomes sexually matured (Radostits et al. 2007). Besides, higher prevalence of brucellosis in older cattle can be attributed to the constant exposure of the cattle over time to the agent.

Very high seropositivity (33.3 %) was observed in cows which gave birth above 2 years interval in the current study. This is supported by earlier reports from Ethiopia (Musa et al. 1990; Hileselassie et al. 2008). The possible reason could be the effects of the disease on reproductive tract causing retained fetal membrane that usually leads to uterine infection and hence poor conception rate.

The present study also revealed the existence of strong association between number of services per conception and seropositivity of brucellosis. This finding is in agreement with a previous study (Bekele et al. 1999). The number of services per conception increases when cattle are repeatedly experience abortion, retained fetal membrane, dystocia and other reproductive health problems.

Likewise, a statistically significant association between pregnancy and seropositivity of *Brucella* was observed in this study. This is in line with a previous report from Ethiopia (Hileselassie et al. 2008). This could be explained by the fact that, erythritol sugar in the placenta and fetal fluid is elevated during gestation period. This stimulates the growth and multiplication of the bacteria in the reproductive organs (Walker 1999). The bacterial load often reduced in months following calving and abortion until the next pregnancy (Coetzer and Tustin 2004; Radostits et al. 2007).

In epidemiological studies, the use of two tests applied serially is recommended to maximize the accuracy of test results (Godfroid et al. 2002). A combination of RBT and c-ELISA or CFT tests is the most widely used serial testing scheme. RBT is highly sensitive test and could easily applied in field conditions whereas, c-ELISA is highly specific usually used as a confirmatory test method (Samui et al. 2007). The combination of these tests in this study could therefore maximize the accuracy of the findings.

Brucellosis is continues to be a major public and animal health problem in Ethiopia. However, there is no national control scheme in place because of economic seasons and lack of information about the disease status at national level (Megersa et al. 2011; Yohannes 2013). Therefore, it is necessary to devise and implement an integrated disease control approach involving inter-sectorial collaborative strategies between animal and human health sectors, nongovernmental and governmental institutions to minimize the burden of the disease.

## Conclusions

It is evident that brucellosis is endemic at low prevalence level in Ethiopia. In this study, we found a very low level seroprevalence at individual animal level in both extensive and intensive farms. However, a high herd level prevalence was recorded in both types of farms; indicating the wide spread distribution of the disease in the area. Factors such as age, sex, calving interval, number of service preconception and reproductive status were associated with seropositivity of *Brucella* species. This study provides important information on epidemiology of brucellosis in cattle in the study areas and highlights the need for implementation of control measures and raising public awareness on zoonotic transmission of brucellosis.
